# *De Novo* Transcriptome and Small RNA Analyses of Two Amorphophallus Species

**DOI:** 10.1371/journal.pone.0095428

**Published:** 2014-04-23

**Authors:** Ying Diao, Chaozhu Yang, Mi Yan, Xingfei Zheng, Surong Jin, Youwei Wang, Zhongli Hu

**Affiliations:** 1 School of Pharmaceutical Sciences, Wuhan University, Wuhan, P. R. China; 2 College of Life Sciences, Wuhan University, Wuhan, P. R. China; 3 Institute of Konjac, Enshi Academy of Agricultural Sciences, Enshi, P. R. China; 4 Criminal Technical Lab, Nan'an District of Chongqing Public Security Bureau, Chongqing, P. R. China; 5 School of Sciences, Wuhan University of Technology, Wuhan, P. R. China; New Mexico State University, United States of America

## Abstract

Konjac is one of the most important glucomannan crops worldwide. The breeding and genomic researches are largely limited by the genetic basis of *Amorphophallus*. In this study, the transcriptomes of *A. konjac* and *A. bulbifer* were constructed using a high-throughput Illumina sequencing platform. All 108,651 unigenes with average lengths of 430 nt in A. konjac and 119,678 unigenes with average lengths of 439 nt were generated from 54,986,020 reads and 52,334,098 reads after filtering and assembly, respectively. A total of 54,453 transcripts in *A. konjac* and 55,525 in *A. bulbifier* were annotated by comparison with Nr, Swiss-Prot, KEGG, and COG databases after removing exogenous contaminated sequences. A total of 80,332 transcripts differentially expressed between *A. konjac* and *A. bulbifer.* The majority of the genes that are associated with konjac glucomannan biosynthetic pathway were identified. Besides, the small RNAs in *A. konjac* leaves were also obtained by deep sequencing technology. All of 5,499,903 sequences of small RNAs were obtained with the length range between 18 and 30 nt. The potential targets for the miRNAs were also predicted according to the konjac transcripts. Our study provides a systematic overview of the konjac glucomannan biosynthesis genes that are involved in konjac leaves and should facilitate further understanding of the crucial roles of carbohydrate synthesis and other important metabolism pathways in *Amorphophallus*.

## Introduction

*Amorphophallus* (Araceae) comprises more than 170 species mainly distributed in tropical regions in Asia and Africa. For example, 26 species are found in China [1]. Thus far, *Amorphophallus* is the only plant species rich in glucomannan. Glucomannan content in the bulbs of some species is approximately 60% dry weight. Konjac glucomannan (KGM) A is a natural polysaccharide mainly composed of D-glucose and D-mannose connected by β-1,4 glycosidic bonds with a D-glucose to D-mannose molecular ratio of 1∶1.6 to 4.2 [2]. KGM is a type of gum with low concentration and high viscosity. KGM also exhibits several unique physical and chemical properties; furthermore, this substance is used as important raw materials in food, pharmaceutical, and chemical industries as well as in agriculture and other fields [3]. *Amorphophallus* contains unsaturated fatty acids, starches, proteins, alkaloids, and amino acids; therefore, this substance is the preferred food of patients with high blood pressure, obesity, diabetes, constipation, colon cancer, and other digestive diseases [4].

As a commercial product, konjac is divided in two types, namely, starch and glucomannan types. The glucomannan type is mainly produced in Asia, where China and Japan are considered as high konjac-cultivating countries. By contrast, wild konjac is cultivated and harvested in Southeast Asia at a small scale. *A. konjac* is the main species cultivated in a relatively large planting area because this species exhibits high yield and good quality of glucomannan. However, *A. konjac* is susceptible to serious diseases particularly to soft rot disease [5]. Furthermore, the resistant gene of *A. konjac* has not been found yet and thus impedes the development of *A. konjac* in crop planting industries. Konjac breeders have found that wild *A. bulbifer* is strongly resistant to disease with a high propagation coefficient and contains relatively high amounts of glucomannan with good quality [6]. *A. bulbifer* has been successfully domesticated and cultivated in Yunnan Province, China because it contains a gene that can be potentially developed.

Till now studies on the genetic basis of *Amorphophallus* are limited. And current data on *Amorphophallus* genome and transcriptome impede the progress of studies on important genes and molecular breeding of this species. The genome size of *Amorphophallus* is relatively large. Among the 14 species of *Amorphophallus*, *A. bulbifer* has a moderate genome size (1C = 9.28 pg), *A. johnsonii* exhibits the largest genome size (1C = 15.83 pg), and *A. prainii* has the smallest genome size (1C = 3.78 pg); in contrast to *Amorphophallus* species, *Oryza sativa* has a genome size of only 0.50 pg/C [7]. Therefore, sequencing the whole genome of *Amorphophallus* species is very difficult. To address this problem, a new high-throughput sequencing technology has also been developed and thus exhibits a revolutionary change in the traditional sequencing method. Sequencing-by-synthesis (SBS)-based Illumina sequencing platform (Illumina) can directly show the read length within 100 bp, in which the relative abundance of specific RNA can be calculated according to the measured frequency of occurrence. This sequencing platform has been widely used in research fields such as functional genomics, cancer and other complex diseases, agricultural resources, and microbiology [8]–[11].

In this study, the Illumina high-throughput sequencing technology and bioinformatics analysis were used to obtain the basic information on transcriptome and small RNAs in *A. konjac* and *A. bulbifer*. These dataset will serve as a public information platform for gene expression, genomics, and functional genomics in *Amorphophallus*.

## Materials and Methods

### Material Preparation

The locations where we collected wild materials were not required a specific permission, because *Amorphophallus konjac* and *Amorphophallus bulbifer* naturally distribute in southeast China, which were also domesticated as a special economic crop. We confirm that the field studies did not involve endangered or protected species. The wild materials of *A. konjac* were collected in Hubei Province, China and *A. bulbifer* were collected in Yunan Province, China. All of them were authenticated by Prof. Zhongli Hu (Wuhan University). The Konjac tubers were planted in jars, separately, and grown in a standard greenhouse at 30/20°C±2 (day/night) with a relative humidity between 70% and 80%. Fresh leaves from five plants of each species were collected and mixed to minimize the effect of transcriptome variability among individual plants, then immediately frozen in liquid nitrogen. Total RNA was extracted with RNeasy (Qiagen, Hilden, Germany).

### mRNA Sequencing

The total RNA was subjected to further analysis. mRNA-seq library construction and sequencing were performed at the Beijing Genomics Institute (BGI) genomic center, Shenzhen, China (http://www.genomics.cn) in accordance with the manufacturer's instructions by using a HiSeq2000 system (Illumina) (San Diego,CA). The data was submitted into the NCBI SRA database (SRA057020). After purity filtering and initial quality tests, the reads were sorted and counted for the following analysis.

### Annotation of the Transcriptome

After removed the adaptors and low-quality reads, the reads with an identity value of 95% and coverage length of 180 bp were assembled using Trinity software, which consists of three modules: Inchworm, Chrysalis and Butterfly [12]. The software first combined reads of certain lengths of overlap to form longer fragments called Contigs. Then, the reads were mapped back to the Contigs, which were connected until extended on neither end. The obtained sequences were defined as Unigenes after removing any redundancy.

These UniGenes were submitted to protein databases for homolog and annotation comparison by BLASTX algorithm (evalue ≤1e-5), including Nr, Swiss-Prot, KEGG, and COG. The GO annotation and functional were analyzed by using Blast2GO [13] and WEGO [14] software. BLASTN was used in the Nt nucleotide database. ESTScan software (http://www.ch.embnet.org/software/ESTScan.html) located the position of the Unigene sequences which were unaligned to the previously mentioned databases [15].

### Identification of differentially expressed genes

The gene expression level was normalized to the values of RPKM (Reads Per kb per Million reads) [16]. Differentially expressed genes (DEGs) between two materials were identified based on a rigorous algorithm developed by Audic and Claverie [17]. A “False Discovery Rate(FDR)≤0.001 and the absolute value of log2-Ratio≥1” was set as the threshold to determine the significance of gene expression difference. The DEGs were also analyzed in GO and KEGG database.

### Small RNAs sequencing

The small RNA fragments of 18–30 nt were isolated from the total RNAs and purified, after 15% denaturing polyacrylamide gel electrophoresis. Then, the small RNAs were ligated to a 5′ and 3′ adaptor sequentially and converted to DNA by RT-PCR. According to the manufacturer's protocols, the reversed products were sequenced directly using Illumina Hiseq 2000,which was performed at Beijing Genomics Institute (BGI), Shenzhen, China. The sequenced short reads data are available at NCBI (data is being uploaded to SRA).

### Small RNA analysis

The sequence data were first get rid of the low quality tags and several inds of contaminants, including incorrect sequencing, adaptor sequences and sequences shorter than 16 nt. The sequences matching non-coding RNAs (rRNA, tRNA, snRNA, snoRNA) available in Rfam (http://www.sanger.ac.uk/software/Rfam) [18] and the GenBank noncoding RNA database (http://www.ncbi.nlm.nih.gov/) were removed. Length distribution of clean reads was then summarized. The remaining reads were used to the further computational analysis. Based on the consensus of the conserved sequence in 5′ end of mature miRNA (called seed region) in the same miRNA family, the known miRNAs in A.konjac was identified from published miRNA datasets (miRBase 14.0) (http://www.mirbase.org/) [19].

### Prediction of miRNA targets

We adopted stringent criteria [20], [21] to predict the potential targets of identified miRNAs. The target sites of miRNAs were predicted by aligning the miRNA sequences with the transcritome data obtained in this study using miRCat (http://srna-tools.cmp.uea.ac.uk/mircat/). The criteria used to predict miRNA targets were as follows: (1) No more than four mismatches between sRNA and the target (G-U bases count as 0.5 mismatches); (2) No more than two adjacent mismatches in the miRNA/target duplex; (3) No adjacent mismatches in positions 2–12 of the miRNA/target duplex (5′ of miRNA); (4) No mismatches in positions 10–11 of the miRNA/target duplex; (5) No more than 2.5 mismatches in positions 1–12 of the miRNA/target duplex (5′ of miRNA); (6) The minimum free energy (MFE) of the miRNA/target duplex should be > = 74% of the MFE miRNA bound to its perfect complement. The functional analysis of the predicted targeted genes was performed by BLASTX searching against KEGG and GO databases.

### Semi-quantitative RT-PCR Analysis

In order to technically validate the data from deep sequencing, five differential expressed unigenes and four miRNA genes were selected for real-time RT-PCR analysis. The specific primers designed with primer premier software (version 5.0) (Table S1). 18S rDNA selected from our transcriptome data was used as the internal reference sequences for unigene analysis and miR-39 downloaded from the database of *Caenorhabditis elegans* (Gene ID: 266867) was for miRNA analysis. Total RNA was extracted from konjac leaves with RNAprep pure Plant Kit (Tiangen, China). First-strand cDNA was synthesized using RevertAid Reverse Transcriptase (Fermentas) and diluted 20 fold as template. Experiments were carried out using all-in-OneTM qPCR Master Mix(GeneCopoeiaTM,AOPR-1200) with StepOne plusTM Real-Time PCR system (Applied Biosystems). Quantifying the relative expression of the genes in two samples was performed using the delta-delta Ct method as described by Livak and Schmittgen [22].

## Results and Discussion

### Results and analysis of transcriptome

#### mRNA sequencing, data processing, and annotation

A total of 54,986,020 reads of *A. konjac* and 52,334,098 reads of *A. bulbifer* were obtained using IlluminaHiSeq 2000 high-throughput sequencing. The sequencing data yields of *A. konjac* and *A. bulbifer* were approximately 4.9 and 4.7 G, respectively. The Q20 ratio (sequencing error rate < 1%); the GC proportions in *A. konjac* were 92.27% and 56.93%, respectively; for *A. bulbifer*, these proportions were 93.48% and 54.29%, respectively (Table 1).

**Table 1:** Summary of the transcriptome of Amorphophallus konjac and Amorphophallus bulbifer.

Sample	Total Reads	Nucleotides (nt)	Total Q20 percentage	N percentage	GC percentage *	Contigs			Unigenes		
						Number	Mean length(nt)	N50	Number	Mean length(nt)	N50
Amorphophallus konjac	54,986,020	4,948,741,800	92.27%	0.00%	56.93%	187459	276	381	108651	439	534
Amorphophallus bulbifer	52,334,098	4,710,068,820	93.48%	0.00%	54.29%	199257	276	372	119678	430	524
All-unigene	-	-	-	-	-	-	-	-	132625	635	635

**Figure 1:**
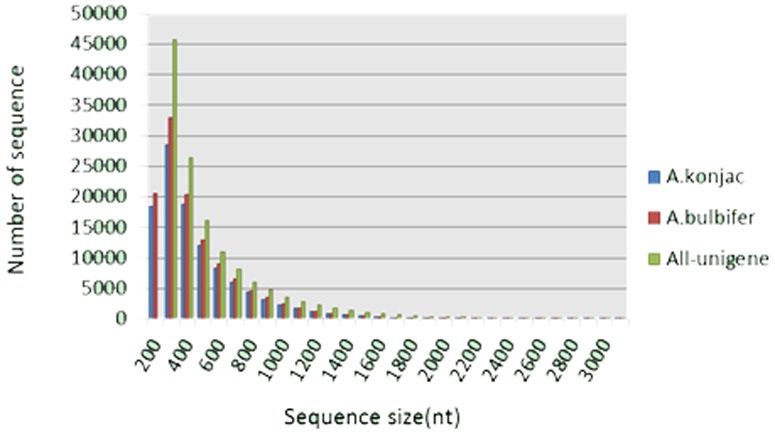
Statistics of unigene assembly qualities. All sizes of the Unigenes were calculated.

**Figure 2:**
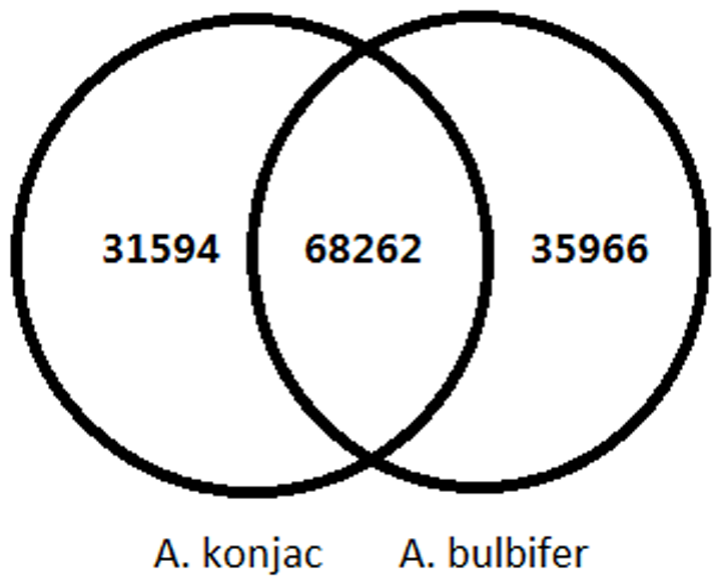
Comparison of the unigenes from *A. konjac* and *A. bulbifer*.

The short reads of *A. konjac* assembled 187,459 contigs and 108,651 unigenes with average lengths of 276 and 430 nt, respectively. The short reads of *A. bulbifer* assembled 199,256 contigs and 119,678 unigenes with average lengths of 276 and 439 nt, respectively. Two EST libraries were merged to assemble the unigene of *Amorphophallus* (defined as all-unigenes). A total of 132,625 all-unigenes with an average length of 523 nt were obtained (Figure 1; Table 1). This comparison indicated that the number of all-unigene sequences longer than 1000 bp was significantly greater than that longer than 1000 bp obtained by a separate assembly in *A. konjac* and *A. bulbifer*. Longer sequences were more favorable for the subsequent bioinformatics analysis. Therefore, the subsequent analysis was mainly based on all-unigenes. The number of transcripts simultaneously found in *A. konjac* and *A. bulbifer* was 65,065. A total of 31,594 and 35,966 all-unigenes were expressed in *A. konjac* and *A. bulbifer*, respectively (Figure 2).

#### Sequence annotation

Sequence alignment using BLAST showed that 63,056 transcripts exhibited gene annotation (Table 2; Table S2). Among these transcripts, 54,453 and 55,525 were found in *A. konjac* and *A. bulbifier*, respectively. The remaining 72,766 transcripts (53.6%), which may be considered as new genes, were not annotated.

**Table 2:** BLAST analysis results against important public databases.

Sample	Number of All-unigenes	NR(%)	Swiss-Prot(%)	COG(%)	GO(%)	KEGG(%)	Total (%)
Amorphophallus konjac	99856	50766(50.8%)	37297(37.4%)	18494(18.5%)	19832(19.9%)	21908(21.9%)	54453 (54.5%)
Amorphophallus bulbifer	104228	51676(49.6%)	38067(36.5%)	18751(18.0%)	20250(19.4%)	22230(21.3%)	55525 (53.3%)
All-unigenes	132625	62194(45.8%)	44706(32.9%)	21610(15.9%)	27773(20.4%)	26079(19.2%)	63056 (46.4%)

**Figure 3:**
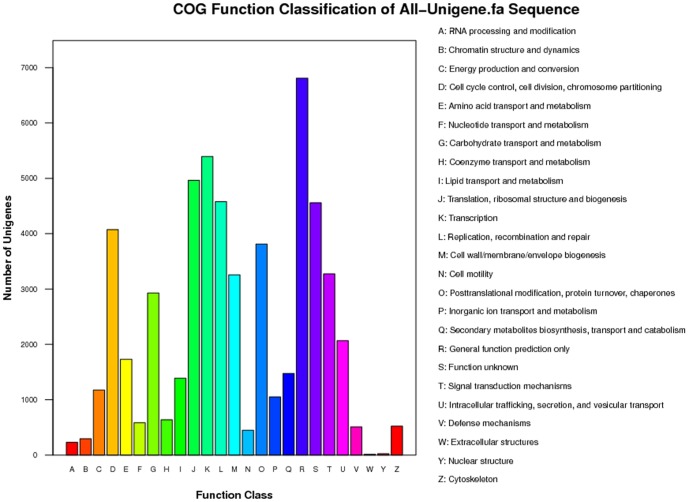
COG function classification of Unigenes.

All-unigenes were aligned to the COG database to predict their possible functions. According to the Nr hits, a total of 21,610 sequences were assigned to 25 categories in the COG database. The cluster of “General function prediction” was the largest group (6,808), followed by “Transcription” (5,393) and “Replication, recombination, and repair” (4,580) groups. Extracellular structures (13 unigenes), nuclear structure (23 unigenes), and RNA processing and modification (231 unigenes) were among the smallest categories (Figure 3).

**Figure 4:**
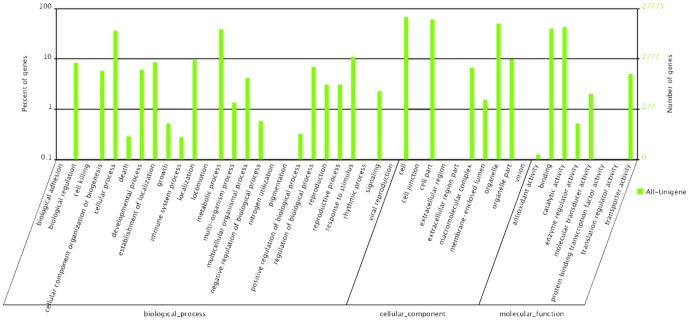
GO categories of the unigenes. The unigenes were annotated in three categories: biological processes, cellular components and molecular functions.

The gene ontology (GO) functional annotation can be obtained according to the Nr annotation information. GO comprises three ontologies that describe molecular functions, cellular components, and biological processes. A total of 27,773 transcripts of *Amorphophallus* were involved in various life activities. In a GO classification system, the three broad categories are molecular function, biological process, and cell components. Among them, 25,109 all-unigenes were involved in molecular functions, 40,810 all-unigenes were involved in biological processes, and 54,943 all-unigenes were involved in cellular components. These broad categories are further divided into 44 small categories, in which the cells (18,876), cell parts (16,991), and organelles (14,036) belonging to the cellular component category include the highest number of genes. Only a few all-unigenes were assigned to virion (2), cell killing (3), nitrogen utilization (3), and translation regulator activity (3) (Figure 4).

To understand the metabolic pathways of *Amorphophallus*, 26,079 all-unigenes were mapped onto 121 paths in the KEGG database (data not shown). The paths containing the largest number of transcripts included “metabolic pathways” (5957), “secondary metabolite biosynthesis” (2789), and “plant pathogen interactions” (1814). The paths containing the least number of transcripts included C5-branched dibasic acid metabolism (10), betalain biosynthesis (9), and fatty acid elongation in the mitochondria (9).

#### Highly expressed transcripts in *Amorphophallus* leaves

The RPKM value corresponding to each transcript represents its expression level. The top 10 transcripts with the highest expression levels in the leaves of *A. konjac* and *A. bulbifer* are listed in Table 3. The results indicated that the transcripts with the highest expression levels in both species were mostly found in photosynthesis-associated structures or enzymes such as photosystems I and II, light-harvesting complex I, and ribulose bisphosphate carboxylase. This result is consistent with the main biological function of the leaves. Interestingly, highly expressed Unigene4984_All was detected in both species. However, the annotation information indicated that this gene corresponds to ORF124 of *Pinus koraiensis*, in which specific biological functions remain unknown. The transcript with the highest expression level in *A. bulbifier* was Unigene103884_All, in which the expression level was 600 times higher than that in *A. konjac*. However, no annotation information on the gene has been found in commonly used databases, indicating that the gene may be specific for *Amorphophallus* and this gene has an important function in *A. bulbifier*.

**Table 3:** High expressed transcripts in A. konjac and A. bulbifer.

Amorphophallus konjac				Amorphophallus bulbifer			
Unigene ID	RPKM	Subject ID	BLAST annotation	Unigene ID	RPKM	Subject ID	BLAST annotation
Unigene22046_All	41264.82	gi|132116|	ribulose bisphosphate carboxylase [Lemna gibba]	Unigene103884_All	24377.54	—	—
Unigene37446_All	35045.81	gi|87621681|	chloroplast chlorophyll a/b binding protein [Pachysandra terminalis]	Unigene28077_All	11149.64	gi|87621681|	chloroplast chlorophyll a/b binding protein [Pachysandra terminalis]
Unigene54425_All	31010.49	gi|115777|	chlorophyl-a/b-binding protein precursor [Silene latifolia subsp. alba]	Unigene54425_All	9442.416	gi|115777|	chlorophyl-a/b-binding protein precursor [Silene latifolia subsp. alba]
Unigene30143_All	12306.43	gi|224100797|	light-harvesting complex I protein Lhca1 [Populus trichocarpa]	Unigene18664_All	9417.155	gi|292559571|	ATP synthase CF0 B chain subunit I [Phoenix dactylifera]
Unigene54241_All	12105.16	gi|255560287|	photosystem I reaction center subunit IV A, chloroplast precursor [Ricinus communis]	Unigene4984_All	8478.411	gi|145408590|	ORF124 [Pinus koraiensis]
Unigene37455_All	10199.83	gi|19855891|	Photosystem I reaction center subunit II, chloroplastic;	Unigene30143_All	7390.616	gi|224100797|	light-harvesting complex I protein Lhca1 [Populus trichocarpa]
Unigene4984_All	9264.685	gi|145408590|	ORF124 [Pinus koraiensis]	Unigene103921_All	7351.332	gi|255560287|	photosystem I reaction center subunit IV A, chloroplast precursor [Ricinus communis]
Unigene26722_All	8563.255	gi|224057740|	light-harvesting complex I protein Lhca2 [Populus trichocarpa]	Unigene22046_All	6429.249	gi|132116|	ribulose bisphosphate carboxylase [Lemna gibba]
Unigene54367_All	8227.831	gi|226358407|	chloroplast chlorophyll A-B binding protein [Gossypium hirsutum]	Unigene104310_All	5445.817	gi|115187527|	photosystem I psaH protein [Arachis hypogaea]
Unigene54476_All	6916.869	gi|255547954|	photosystem II core complex proteins psbY, chloroplast precursor [Ricinus communis]	Unigene87696_All	5429.246	gi|87621681|	chloroplast chlorophyll a/b binding protein [Pachysandra terminalis]

#### Analysis of differentially expressed genes

**Figure 5:**
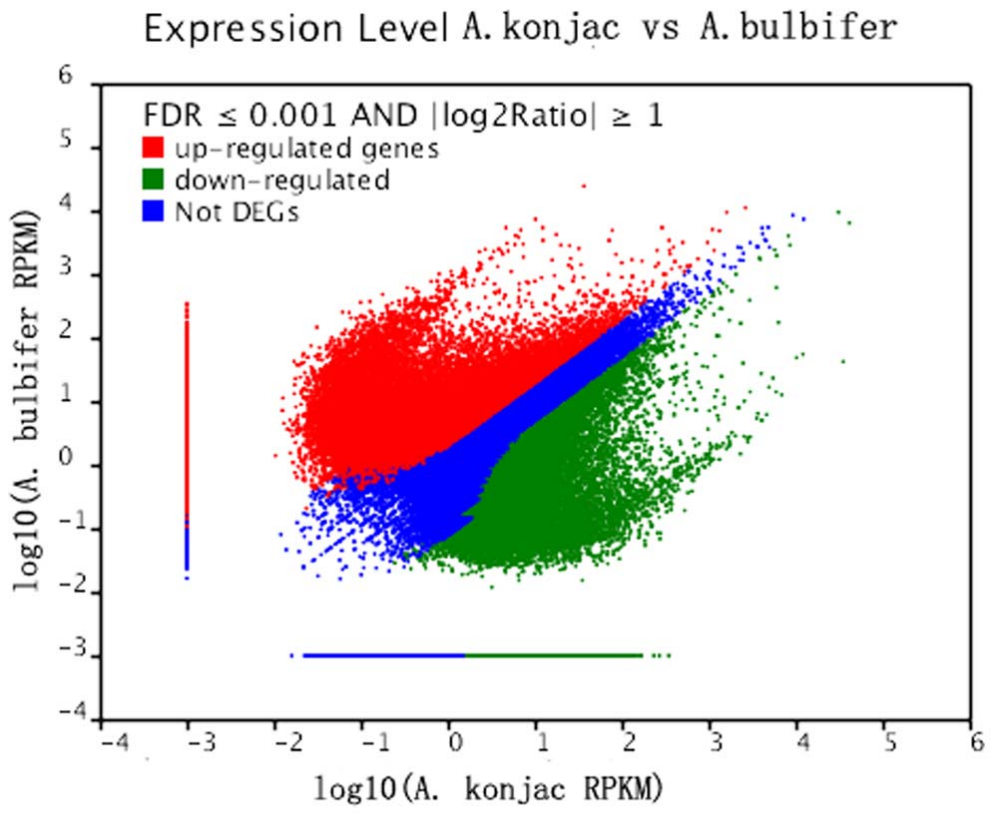
Differentially expressed genes between *A. konjac* and *A. bulbifer.*

We found 80,332 transcripts differentially expressed between the two samples by comparing the expression levels (Table S3; Figure 5). A total of 46,013 all-unigenes were upregulated and 34,319 genes were downregulated in *A. bulbifier* compared with *A. konjac*. A total of 52,293 all-unigenes were expressed without a significant difference in both species.

Among the all-unigenes simultaneously expressed in both species, a total of 36,455 were differentially expressed genes. The genes with the highest levels of upregulation may correspond to the genes encoding bifunctional inhibitor/lipid-transfer protein (Unigene85551_All, |gb| AEE84613.1|) when *A. bulbifier* was compared with *A. konjac*; the two other upregulated genes were Unigene104301_All and Unigene103937_All without relevant annotation information. The downregulated gene corresponded to the protein of the gene encoding photosystem II (Unigene53975_All, gi|113536773|dbj|BAF09156.1|), the two other downregulated genes were Unigene36964_All and Unigene54336_All without relevant annotation information.

GO analysis showed that most differentially expressed genes were attributed to the following: metabolic process (6258) in the biological process category; cell (11,201) in the cellular component category; and catalytic activity (6,990) in the molecular function category (data not shown).

The results of KEGG annotation of differentially expressed genes showed that most of these genes were distributed in metabolic pathways (3587), biosynthesis of secondary metabolites (1758), and plant-pathogen interactions (1013; data not shown).

### Small RNA sequencing and analysis

#### Small RNA sequence information

**Figure 6:**
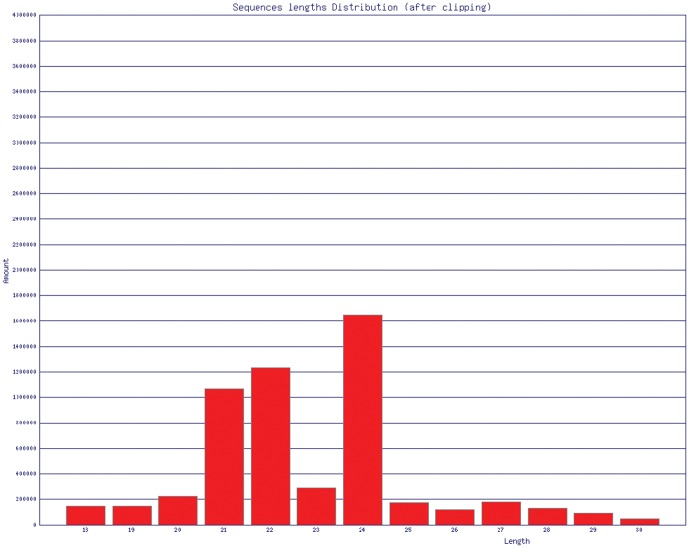
Sequence length distribution of small RNAs.

The sequences of the small RNAs in *A. konjac* leaves were obtained by deep sequencing technology. After the adaptors were removed, 5,499,903 sequences were obtained. The sequence lengths ranged between 18 and 30 nt. The length of the sequences ranging from 20 nt to 24 nt accounted for 4.1%, 19.5%, 22.4%, 5.3%, and 30.0% of the total sequences, respectively (Figure 6). BLAST alignment showed that 1,095,428 sequences were aligned with rfam database (Table 4), in which miRNA exhibited a high proportion of 61.6%; rRNA and tRNA also revealed high proportions of 25.9% and 10.7%, respectively.

**Table 4:** Statistics of small RNAs in Amorphophallus konjac.

category	number	category	number	category	number
Gene;snRNA;splicing;	1362	Cis-reg;riboswitch;	61	Intron;	7653
Gene;antitoxin;	1	Cis-reg;IRES;	7	Cis-reg;thermoregulator;	12
Gene;ribozyme;	44	Gene;snRNA;snoRNA;scaRNA;	5	Cis-reg;leader;	8
Gene;miRNA;	674825	Gene;snRNA;snoRNA;CD-box;	3389	Gene;rRNA;	283314
Gene;antisense;	60	Gene;snRNA;snoRNA;HACA-box;	96	Gene;sRNA;	240
Gene;	909	Cis-reg;	6406	Gene;snRNA;	4
Cis-reg;frameshift_element;	19	Gene;lncRNA;	31	Gene;tRNA;	116982

#### Analysis of conserved miRNAs

The sequence alignment using the miRBase database revealed that 146 conserved miRNAs belonging to 18 miRNA families were expressed in the leaves of *A. konjac*. Among these miRNAs, miR166h (603919), miR166f (603918), and miR166k (602540) belonging to the miR166 family exhibited the highest expression levels, whereas miR4376, miR156i, and miR156g with only one transcript exhibited the lowest expression levels. The statistical information of known conserved miRNAs is shown in Table S4. The study also found that the expression levels of various members in the same miRNA family differed. For instance, the difference between members was more significant than that between this miRNA family and another miRNA family. For example, the expression level of miR166u was only 363, which was less than the average expression levels of the other members of this miRNA family.

#### Target gene prediction

The target genes were predicted based on the characteristics of high complementarity of miRNAs with the target gene sequence. The results showed that 1197 transcripts of *A. konjac* were the potential target genes of miRNAs. Some target genes exhibited no definite functions, while the annotated targets are involved in transcriptional regulation, metabolism, signal transduction, stress response, electronic transmission, and other life processes (Table S5).

Few studies on the regulation of glucomannan synthesis have been performed. Through conducting miRNA target gene prediction, we found that four members of the genes participating in glucomannan synthesis are possibly regulated by miRNA. These members were SS (Unigene21839_All) and corresponding miR339, UGP (Unigene28076_All) and corresponding miR156 and starch synthase III precursor (Unigene18245_All) and corresponding miR5763. These miRNA may perform important regulatory functions in KGM synthesis. For example, miR339 induces the silencing of sucrose synthase mRNA by combining with the transcripts of sucrose synthase, thereby controlling sucrose degradation and sucrose synthase production at a transcription level. This process also regulates starch and glucomannan synthesis. In the leaves, fructose and glucose can be obtained via sucrose decomposition and also can be directly produced by photosynthesis, providing raw materials of glucomannan and starch synthesis. However, fructose and glucose in the corms of Amorphophallus are mainly obtained from sucrose decomposition. Therefore, the suppression of sucrose synthase in the leaves possibly enhanced the use of photosynthesis-produced glucose and fructose in starch and glucomannan synthesis.

### Gene validation and expression analysis

**Figure 7:**
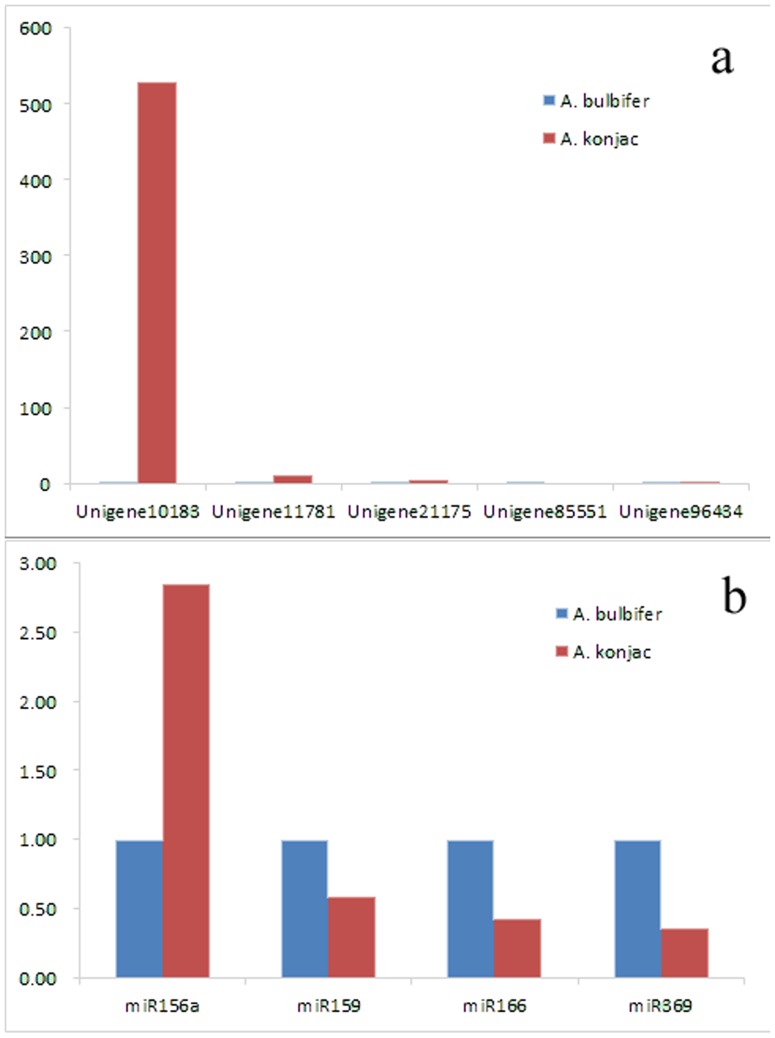
Validation of candidate unigenes and miRNAs in *A. konjac* and *A. bulbifer* by qRT-PCR. (a) Five candidate unigenes show differential expression patterns by qRT-PCR in *A. konjac* and *A. bulbifer*. (b) Four candidate miRNAs show differential expression patterns by qRT-PCR in *A. konjac* and *A. bulbifer*.

According the data from deep sequencing, five selected unigenes were differential expressed between *A. konjac* and *A. bulbifer*. Unigene21175_All, Unigene11781_All and Unigene10183_All were expressed much higher in *A. konjac* than in *A. bulbifer*, while the expression levels of Unigene96434_All and Unigene85551_All were much lower in *A. konjac* than in *A. bulbifer*. As the results of real-time RT-PCR shown in Figure 7a, the expression patterns of all detected genes show the same trend using RT-PCR and the Solexa-sequencing method. The expression of the four miRNAs identified by Solexa sequencing in *A. konjac* was assayed using qRT-PCR analysis and signals were detected in both of the two *Amorphophallus* species (Figure 7b). Therefore, these miRNAs are authentic miRNAs. As shown in the figure, these miRNAs were expressed in differential levels between the two samples. Four miRNAs were expressed higher in *A. bulbifer* than in *A. konjac*, except miR156a were expressed much lower in *A. bulbifer* than in *A. konjac*.

In sum, these results suggest that Solexa sequencing is an accurate and efficient technique to discover both transcripts of genes and miRNAs from *Amorphophallus* species.

### Construction of KGM biosynthetic pathway

**Figure 8:**
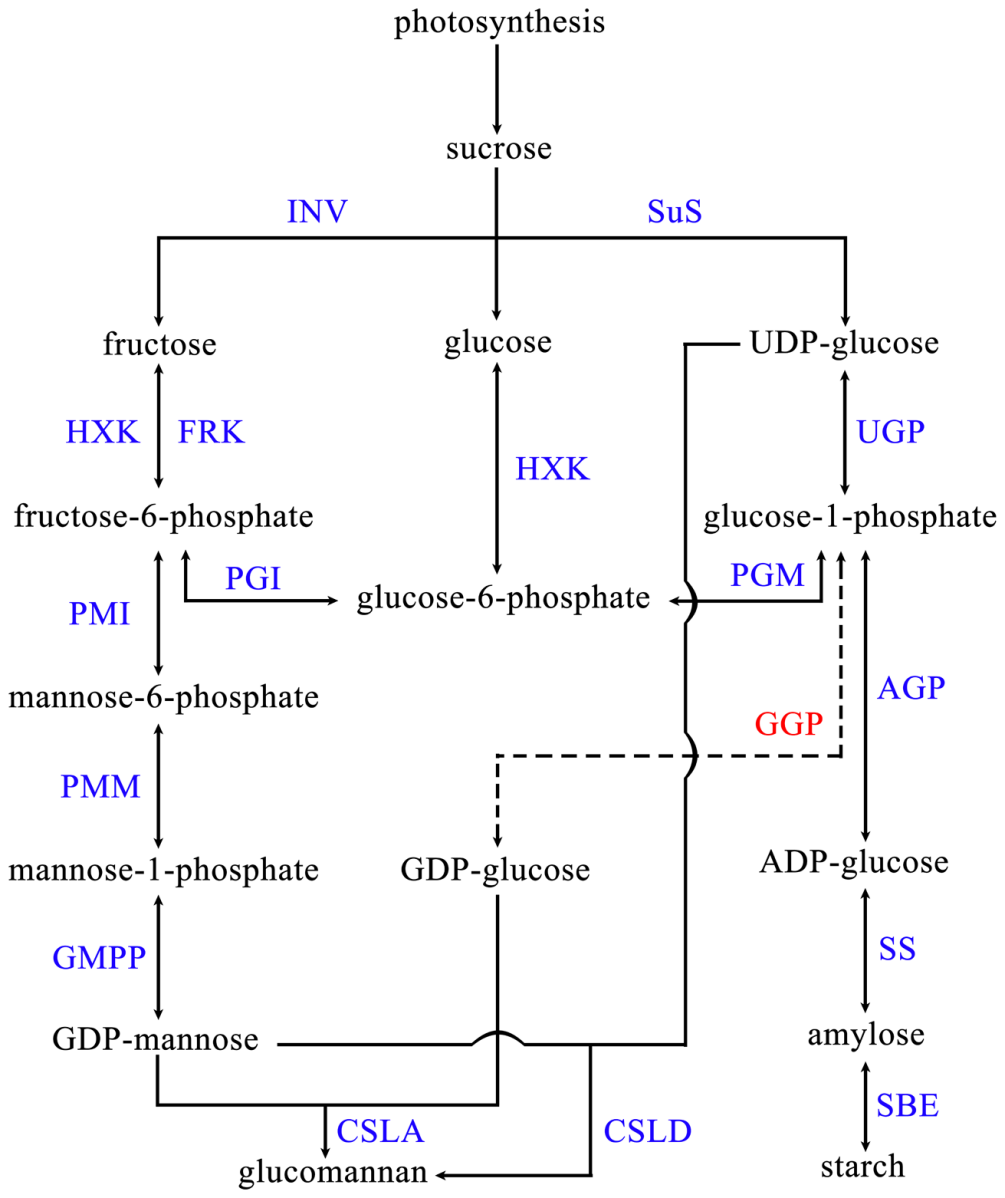
Proposed pathways of konjac glucomannan biosynthesis. The identified enzymes in *Amorphophallus* are noted in blue and the unidentified is in red. Sucrose synthase (SuS), invertase (INV), phosphoglucose isomerase (PGI), phosphoglucomutase (PGM), phosphomannose isomerase (PMI), phosphomannomutase (PMM), starch synthase (SS), GDP-mannose pyrophosphorylase (GMPP), UDP-glucose pyrophosphorylase (UGP), ADP-glucose pyrophosphorylase (AGP), fructokinase (FRK), hexokinase (HXK), starch branching enzyme (SDB), cellulose synthase-like A (CSLA), Cellulose synthase-like D (CSLD), GDP-D-pyrophosphorylase (GGP)

The carbohydrates in *Amorphophallus* contain relatively complex substances such as glucose, fructose, starch, sucrose, and glucomannan. Among these substances, fructose and glucose are synthesized via photosynthesis in daytime or obtained from sucrose degradation, in which sucrose is converted to produce starch or glucomannan and other polysaccharides. So, the pathway of glucomannan biosynthesis was related to sucrose metabolism, nucleotide sugar conversion pathways. Six of these enzymes, namely, sucrose synthase (SuS), phosphoglucose isomerase (PGI), phosphoglucomutase (PGM), phosphomannose isomerase (PMI), phosphomannomutase (PMM), and starch synthase (SS) are found in *Amorphophallus*. Experimental evidence has also shown that these enzymes exhibit corresponding catalytic functions [23]. Furthermore, AkCSLA3 gene was cloned from *A. konjac* and the enzyme was confirmed having the glucomannan mannosyl- and glucosyl transferase activities [24]. The structure and phylogeny of CSLD proteins have led to suggestions that the proteins would be glucan synthases using UDP-glucose as a substrate and preliminary research has indicated that CSLD proteins are also glucomannan synthases [25]. Considering the annotation results of the transcriptome data and the reported results, the possible biosynthetic pathway of KGM and starch in konjac leaf was constructed (Fig. 8, Table 5, Table S6). Among these transcripts, the mRNA sequences of fructokinase (FRK) and cellulose synthase-like D (CSLD) were reported for the first time, indicating that the corresponding genes of the two enzymes were present in *A. konjac* and *A. bulbifer*. Nevertheless, enzyme activities should be confirmed by conducting further investigations. In the known glucomannan biosynthesis pathways, only GDP-D-pyrophosphorylase (GGP) is absent in the leaves of *Amorphophallus*. And GGP is also not found in konjac corms [24]. Heller et al. (1972) found UDP-glucose, ADP-glucose and GDP-mannose in konjac corms, but no GDP-glucose [26]. It seems there is little probability of the glucose units in KGMs obtained from GDP-glucose. The possible way of KGM synthesis might be GDP-mannose and UDP-glucose was catalyzed by CSLD proteins. This deduction needs the further experiments.

**Table 5:** Statistics of Glucomannan and starch biosynthesis related genes in Amorphophallus.

Enzyme	Symbol	Number of EC	Annotation	Count of Unigenes in A. konjac	Count of Unigenes in A. bulbifer
Sucrose synthase	SuS	2.4.1.13	sucrose synthase	18	19
Invertase	INV	3.2.1.26	Beta-fructosidases	13	12
Hexokinase	HXK	2.7.1.1	hexokinase	14	14
Fructokinase	FRK	2.7.1.4	fructokinase	9	12
Phosphoglucose isomerase	PGI	5.3.1.9	Glucose-6-phosphate isomerase	1	1
Phosphomannose isomerase	PMI	5.3.1.8	Mannose-6-phosphate isomerase	2	2
Phosphomannomutase	PMM	5.4.2.8	Phosphomannomutase	3	2
GDP-mannose pyrophosphorylase	GMPP	2.7.7.13	GDP-mannose pyrophosphorylase	2	2
		2.7.7.22	GDP-mannose pyrophosphorylase	1	1
Phosphoglucomutase	PGM	5.4.2.2	Phosphoglucomutase	9	12
ADP-glucose pyrophosphorylase	AGP	2.7.7.27	glucose-1-phosphate adenylyltransferase	21	22
UDP-glucose pyrophosphorylase	UGP	2.7.7.9	UTP—glucose-1-phosphate uridylyltransferase	8	9
Starch synthase	SSS	2.4.1.21	Soluble starch synthase	21	18
	GBSS	2.4.1.242	Granule-bound starch synthase	2	4
	SDE	3.2.1.-	starch debranching enzyme	2	2
Starch branching enzyme	SBE	2.4.1.18	1,4-alpha-glucan branching enzyme	11	11
Cellulose synthase-like A	CSLA	2.4.1.32	Cellulose synthase-like A2	3	3
			Cellulose synthase-like A3	3	3
			Cellulose synthase-like A9	16	18
Cellulose synthase-like D	CSLD	2.4.2.24	Cellulose synthase-like D2	5	6
			Cellulose synthase-like D3	1	1
			Cellulose synthase-like D5	1	2

In the expressed genes, the numbers of the corresponding transcripts of various functional genes differed significantly. For example, PGI exhibited only one type of transcript with a significantly higher expression level in *A. bulbifier* than in *A. konjac*. PMI showed only two types of transcripts with a significantly lower expression level in *A. bulbifier* than in *A. konjac*.

As GDP-mannose is synthesized from mannose-1-phosphate, two types of GDP-mannose pyrophosphorylase (GMPP) are involved based on the substrate type: type I (EC2.7.7.13) uses GTP and mannose-1-phosphate as the substrates and type II (EC 2.7.7.22) uses GDP and mannose-1-phosphate as the substrates [27], [28]. In this study, the corresponding transcripts of both types of enzymes were present in *Amorphophallus* leaves, but type II revealed only one transcript with a low expression, indicating that GMPP type I was the main enzyme involved in the catalytic synthesis of GDP-mannose.

The starch is categorized into amylose and amylopectin. The synthesis of plant amylose is catalyzed by granule-bound starch synthase (GBSS); amylopectin synthesis can be synergistically catalyzed by soluble starch synthase (SSS), starch branching enzyme (SBE), and debranching enzyme (DBE) [29], [30]. The corresponding transcripts of these four enzymes localized in the chloroplasts were found in the transcriptome of *Amorphophallus*. GBSS exhibited fewer transcripts but higher expression levels. SSS and SBE showed higher numbers of transcripts. Significant differences in expression levels were observed between various transcripts. DBE comprised only two transcripts with relatively low expression levels.

The KGM content is an important indicator of quality. Therefore, the starch content of *Amorphophallus* affects the glucomannan content. In glucomannan and starch synthesis pathway, glucose-1-phosphate is catalyzed to synthesize GDP-glucose, ADP-glucose or DUP-glucose, which participate in glucomannan synthesis or starch synthesis, respectively. But no evidence revealed GGP existing in konjac. Therefore, UGP and AGP are the key enzymes used to determine the in vivo synthesis of glucomannan and starch in *Amorphophallus*. AGP is composed of two subunits, a large subunit and a small subunit; the small subunit is involved in catalytic reactions [31]–[33]. In the same sample, the small subunit of AGP contained three transcripts; the longest transcript exhibited the highest expression level. Furthermore, the expression level of this unigene in *A. bulbifier* was significantly lower than that in *A. konjac*. Among the UGP transcripts, only two transcripts showed equal expression levels in *A. bulbifier* and *A. konjac*. By contrast, the expression levels of the other transcripts in *A. bulbifier* were significantly higher than those in *A. konjac*. At a transcriptional level, *A. bulbifier* exhibited fewer AGP gene transcripts and more UGP gene transcripts than *A. konjac*. Therefore, the amount of glucose-1-phosphate determines whether the pathway is either starch synthesis or glucomannan synthesis. In particular, greater amounts of glucose-1-phosphate in *A. bulbifier* than in *A. konjac* corresponded to glucomannan synthesis. The results also showed that *A. bulbifier* contained higher glucomannan than *A. konjac*.

## Conclusions

Deep RNA sequencing technique can be used to investigate known and unknown transcription information from numerous sources. For conserved genes, the expression conditions in different materials can be indirectly indicated by abundance analysis. This study analyzed the transcriptome data and found the potential genes involved in the biosynthetic pathway of KGM and miRNAs involved in regulation. Furthermore, the overall comparison of the transcriptome data showed that the gene composition and gene expression of *A. konjac* significantly differed from those in *A. bulbifer*. In addition, numerous unknown genes were present in *Amorphophallus*. Our results about transcriptomes and small RNAs could help investigate large amounts of important functional genes in *Amorphophallus* rapidly and effectively to promote the studies on the molecular genetics of *Amorphophallus*.

## Supporting Information

Table S1 **List of primers used for RT-PCR.**
(XLSX)

Table S2 **Summary of unigene annotation.**
(ZIP)

Table S3 **Differentially expressed unigenes.**
(XLSX)

Table S4 **Statistic of conserved miRNAs.**
(XLSX)

Table S5 **Annotation of the potential target genes.**
(XLSX)

Table S6 **Putative genes in KGM and starch metabolism.**
(XLSX)
